# Clinical Reasoning in Veterinary Ophthalmology: A Pathophysiology-Centered Framework for Canine and Feline Clinical Practice

**DOI:** 10.3390/ani16203185

**Published:** 2026-10-10

**Authors:** Nobuyuki Kanemaki

**Affiliations:** 1 School of Veterinary Medicine, Azabu University, 1-17-71 Fuchinobe, Chuo-ku, Sagamihara 252-5201, Kanagawa, Japan; kanemaki@azabu-u.ac.jp; Tel.: +81-45-320-9099; 2 DVMs Animal Medical Center Yokohama, 2-2 Sawatari, Kanagawa-ku, Yokohama 221-0844, Kanagawa, Japan

**Keywords:** cats, clinical decision-making, clinical reasoning, dogs, pathophysiology-centered reasoning, theoretical frameworks, veterinary ophthalmology

## Abstract

Clinical reasoning enables veterinarians to make clinical decisions for individual patients by integrating clinical information with scientific evidence, knowledge of underlying disease processes, and case-specific circumstances. This review aimed to clarify clinical reasoning in canine and feline ophthalmology, with particular emphasis on pathophysiological interpretation, and to organize major theoretical frameworks of clinical reasoning in relation to the ophthalmic diagnostic process. A narrative and conceptual review was conducted using PubMed and Google Scholar, with literature searched from February 2026 through 31 August 2026. Relevant publications were selected according to their conceptual contribution to clinical reasoning, cognition, decision-making, clinical practice, or clinical education rather than predefined systematic-review eligibility criteria. Fifteen theoretical frameworks were organized into four groups according to their primary theoretical roles: historical and normative theories of reasoning; foundational theories of modern cognitive science; theories of clinical reasoning and decision-making; and theories of clinical education and expertise. The framework was illustrated through three representative clinical scenarios involving glaucoma in a Shiba Inu, a descemetocele with aqueous humor leakage in a Japanese cat, and traumatic proptosis in a Chihuahua. The analysis demonstrates how different theoretical perspectives can contribute to the interpretation of clinical information and its connection to pathophysiological understanding and clinical decision-making. PCCR is proposed as an integrative clinical reasoning framework that places pathophysiological interpretation between clinical information and patient-specific decision-making. Its applicability beyond veterinary ophthalmology remains a conceptual proposition requiring future evaluation.

## Simple Summary

Veterinarians must make clinical decisions for individual animals based on information obtained from clinical examinations, while also considering scientific evidence, patient-specific circumstances, and animal welfare. This review examines how clinical information can be interpreted in relation to underlying biological processes during clinical decision-making. Fifteen theoretical frameworks of clinical reasoning were organized into four groups according to their primary theoretical roles: historical and normative theories of reasoning; foundational theories of modern cognitive science; theories of clinical reasoning and decision-making; and theories of clinical education and expertise. Their characteristics were illustrated through three representative ophthalmic scenarios involving glaucoma in a Shiba Inu, a descemetocele with aqueous humor leakage in a Japanese cat, and traumatic proptosis in a Chihuahua. The proposed pathophysiology-centered clinical reasoning (PCCR) framework is intended as an integrative clinical framework rather than a new cognitive theory. Although illustrated in veterinary ophthalmology, its applicability to other areas of veterinary medicine remains a hypothesis requiring further evaluation.

## 1. Introduction

In 2013, Stephen A. May of the Royal Veterinary College (UK) explicitly addressed the importance of clinical reasoning and case-based decision-making in veterinary education, identifying the development and teaching of reasoning skills required for clinical practice as a fundamental challenge for veterinary educators [1]. Subsequent studies have examined the development of clinical reasoning in veterinary students and its integration into veterinary curricula [2,3]. Vinten et al. identified clinical reasoning as a fundamental skill for veterinary clinicians and highlighted challenges in its development within veterinary curricula, while Humm and May demonstrated that, although the importance of clinical reasoning was recognized by students and clinicians, opportunities to explicitly promote and practice these skills in first-opinion practice were limited [2,3]. Clinical reasoning has therefore increasingly been recognized as an important component of veterinary clinical competence and as a skill that should be developed before graduation [2,4].

These developments emerged in the context of earlier educational approaches, including problem-oriented medical records (POMRs) and problem-based learning (PBL), which had already been incorporated into veterinary education. POMRs provided a structured approach to organizing clinical information around identified problems and associated assessment and management plans, whereas PBL promoted active, case-based learning and problem solving [5,6,7]. Although these approaches made important contributions to structured clinical problem solving and active learning, they were not, in themselves, comprehensive frameworks for explicitly teaching the cognitive processes underlying clinical reasoning and diagnostic decision-making. Consequently, the development of diagnostic expertise was often embedded within the broader acquisition of knowledge and clinical experience, with students progressively learning to recognize and manage clinical problems [2,3].

May’s work helped articulate the need to make clinical reasoning an explicit educational objective in veterinary education. Rather than focusing solely on identifying the correct diagnosis, increasing attention has been directed toward how clinicians acquire and interpret clinical information, generate and evaluate diagnostic hypotheses, recognize patterns, and arrive at appropriate clinical decisions [1]. Clinical reasoning therefore represents not simply the acquisition of diagnostic knowledge, but the cognitive process through which clinical information is interpreted and transformed into clinically meaningful decisions. Recent educational literature similarly emphasizes the need to make clinical reasoning explicit, structured, and teachable rather than leaving it entirely implicit within clinical experience [8,9].

Clinical reasoning is not a single cognitive mechanism. Rather, it encompasses multiple forms of reasoning and decision-making and is informed by concepts derived from logic, cognitive science, cognitive psychology, and clinical education. Understanding how these theoretical perspectives contribute to clinical reasoning is therefore essential for clarifying how clinicians interpret complex clinical information and make decisions in practice. At the same time, these theoretical perspectives address different aspects of clinical reasoning and may overlap conceptually rather than constituting mutually exclusive processes.

In veterinary ophthalmology, this issue is particularly relevant because ophthalmic diagnosis depends not only on recognizing ocular lesions but also on interpreting their anatomical, physiological, and pathophysiological relationships. A comprehensive ophthalmic examination provides a structured collection of clinical information; however, the clinical significance of individual findings only emerges when these findings are integrated into a coherent representation of the underlying disease process. Pathophysiological interpretation is therefore a central component of clinical reasoning in veterinary ophthalmology, linking observed clinical findings to mechanisms of disease and, ultimately, to patient-specific clinical decision-making.

**Figure 1:**
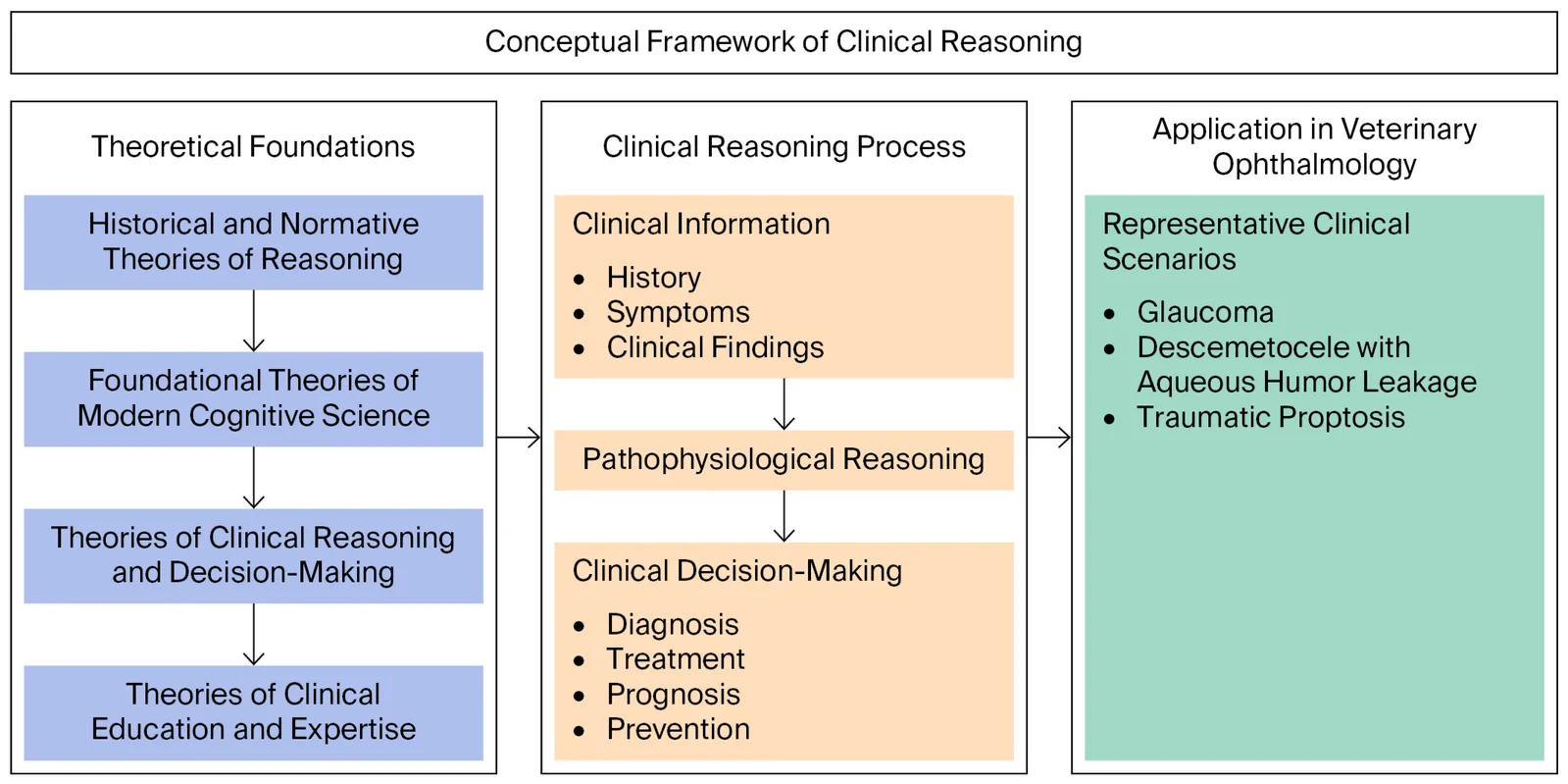
Conceptual framework illustrating the clinical reasoning process and its theoretical foundations in canine and feline clinical practice.

On this basis, this review proposes a pathophysiology-centered clinical reasoning (PCCR) framework for veterinary ophthalmology. As illustrated in Figure 1, the framework places pathophysiological interpretation between the acquisition and organization of clinical information and subsequent patient-specific clinical decision-making. The framework is examined through three representative clinical scenarios in dogs and cats: glaucoma, descemetocele with aqueous humor leakage, and traumatic proptosis. These scenarios were selected because they illustrate different patterns of clinical information, pathophysiological interpretation, and clinical decision-making, thereby providing practical contexts in which the theoretical foundations of clinical reasoning can be examined. The proposed framework is intended as an integrative clinical framework rather than as a new cognitive theory. Its purpose is to make explicit the role of pathophysiological interpretation in connecting clinical information with assessment and patient-specific clinical decision-making.

The aim of this review is to clarify the theoretical foundations of clinical reasoning and to examine how pathophysiological interpretation can connect clinical information with patient-specific clinical decision-making in canine and feline ophthalmology. The framework is illustrated through representative ophthalmic scenarios and considered in relation to contemporary developments in veterinary education and diagnostic technology.

## 2. Materials and Methods

### 2.1. Literature Search and Selection

This review was conducted as a narrative and conceptual review of the theoretical foundations and clinical application of clinical reasoning in veterinary ophthalmology. The literature search was conducted primarily using PubMed from February 2026 through 31 August 2026. Google Scholar was additionally used to identify accessible full-text versions when relevant publications identified through PubMed were not readily accessible. Search terms were developed iteratively according to the major conceptual domains addressed in the review, including clinical reasoning, diagnostic reasoning, clinical decision-making, cognitive science, cognitive psychology, logic, veterinary education, pathophysiological reasoning, and veterinary ophthalmology.

Relevant publications were selected according to their conceptual contribution to the objectives of the review. Core literature was identified from influential or foundational publications describing major theories of reasoning, cognition, clinical reasoning and decision-making, clinical education, and veterinary clinical reasoning. Additional publications were identified from reference lists of relevant articles and from established academic literature in veterinary medicine, human medicine, cognitive science, and clinical education. Conceptual relevance was defined as a publication’s contribution to understanding one or more of the following: (1) the organization and interpretation of clinical information; (2) inference, hypothesis generation, or decision-making under uncertainty; (3) pathophysiological interpretation of individual patient findings; (4) clinical reasoning in practice; or (5) the acquisition, monitoring, or development of clinical reasoning expertise. Because this was a narrative and conceptual review, literature was not selected according to predefined systematic-review eligibility criteria, and the search was not intended to be exhaustive.

The 15 theoretical frameworks discussed in this review were selected because they represent major conceptual perspectives relevant to these domains and because they provide complementary explanations of different dimensions of clinical reasoning. The four categories were defined according to the primary theoretical role of each framework rather than as mutually exclusive taxonomic groups. Frameworks were not included simply because they addressed clinical reasoning; they were included when their principal concepts contributed to the conceptual synthesis developed in this review. Other theories and models of clinical reasoning may therefore be relevant but were outside the scope of the present synthesis. The categories are intended to organize the literature for conceptual clarity rather than to imply that the selected frameworks constitute an exhaustive or universally accepted classification.

Full-text articles were obtained through the institutional network of Azabu University when required. The retrieved literature was organized and managed using Zotero (version 9.0.6).

### 2.2. Literature Verification and Reference Management

The author reviewed the original publications underlying the principal theoretical concepts and clinical claims discussed in this review. Particular attention was given to primary or authoritative sources describing major theories of reasoning, decision-making, cognitive science, clinical education, and veterinary clinical reasoning.

The correspondence between individual claims and their cited sources was also verified by the author, who independently checked the original publications before inclusion in the manuscript.

The author was responsible for the final selection, interpretation, and citation of all literature included in the review.

### 2.3. Clinical Cases Used in the Representative Scenarios

Two dogs and one cat included in the representative clinical scenarios were treated at DVMs Animal Medical Center Yokohama, where the author serves as a veterinary ophthalmologist. The owners of the animals provided informed consent for the academic use and publication of the clinical information and photographs presented in this review.

## 3. Clinical Reasoning Process

Clinical reasoning can be understood within the broader context of clinical practice. Clinical practice encompasses the cognitive and practical activities performed by veterinarians to identify, diagnose, treat, and monitor a patient’s condition. It begins with gathering information through the medical history and physical examination, followed by identifying clinical problems, interpreting pathophysiological processes through clinical reasoning, formulating diagnostic and therapeutic plans, implementing treatment, and continuously reassessing the patient’s response and prognosis.

The problem-oriented medical record (POMR) approach, proposed by Weed (1968) [10], together with the SOAP (subjective, objective, assessment, and plan) format, represents more than a system for documenting medical information. Based on the concept of organizing medical care around individual problems, the POMR provides a structured approach to organizing clinical information, diagnostic reasoning, and patient management [11,12]. The POMR approach consists of four major components: the database, problem list, initial plan, and progress notes, with SOAP providing a structured format for documenting progress notes. The problem-oriented approach has since been widely adopted in veterinary education and clinical practice.

Subsequently, educational approaches designed to develop clinical problem-solving skills—including problem identification, differential diagnosis, diagnostic testing, and diagnostic decision-making—became increasingly incorporated into veterinary curricula [6,13,14,15]. During the 1990s, evidence-based medicine (EBM) emerged in human medicine to promote clinical decision-making informed by the best available evidence. The concept subsequently developed in veterinary medicine as evidence-based veterinary medicine (EBVM), with the publication of the first dedicated EBVM textbook in 2003 helping to formalize the field [16,17,18,19].

Unlike human medicine, veterinary clinical decision-making must account for additional contextual factors, including animal welfare, client preferences and expectations, economic constraints, and species-specific considerations. Veterinary practitioners also make decisions in clinical contexts in which the available evidence may be limited or heterogeneous [19,20,21].

Accordingly, EBVM provides a framework for identifying, appraising, and applying the best available scientific evidence, whereas clinical reasoning addresses how veterinarians interpret clinical information and integrate it with patient- and case-specific considerations. Clinical reasoning has subsequently been recognized as an important component of veterinary medical education and competency-based training [1,2,8]. Contemporary Day One competency frameworks explicitly include clinical reasoning and decision-making as core areas of veterinary graduate competence.

For the purposes of this review, clinical reasoning is conceptualized as a series of interconnected processes, including (1) information gathering, (2) problem formulation, (3) differential diagnosis, (4) pathophysiological reasoning, (5) selection of diagnostic tests, (6) diagnosis, (7) treatment planning, and (8) follow-up and reassessment [1,2,8]. These processes are not necessarily sequential; rather, they interact dynamically as new information becomes available and clinical hypotheses are revised. Furthermore, clinical reasoning can be understood from several theoretical perspectives, each of which addresses a different aspect of the reasoning process. These perspectives encompass the processes involved in acquiring, organizing, and interpreting clinical information; the cognitive processes involved in clinicians’ reasoning and decision-making; the processes through which reasoning is enacted in practical clinical settings; and the educational perspectives that support the development of clinical reasoning skills.

Among these processes, pathophysiological reasoning is positioned in this review as a central component of clinical reasoning. Through repeated clinical experience, pathophysiological knowledge becomes progressively organized and encapsulated into clinically meaningful concepts, enabling experienced clinicians to recognize disease patterns without consciously recalling individual pathophysiological facts [22,23,24]. This cognitive organization supports efficient clinical decision-making regarding diagnosis, treatment, prognosis, and prevention. Pathophysiological reasoning provides a mechanistic link between morphological and functional abnormalities and their clinical manifestations, thereby supporting these clinical decisions. To accomplish this, veterinarians must accurately interpret information obtained from medical history, physical examination, and diagnostic tests while avoiding unnecessary data collection.

**Figure 2:**
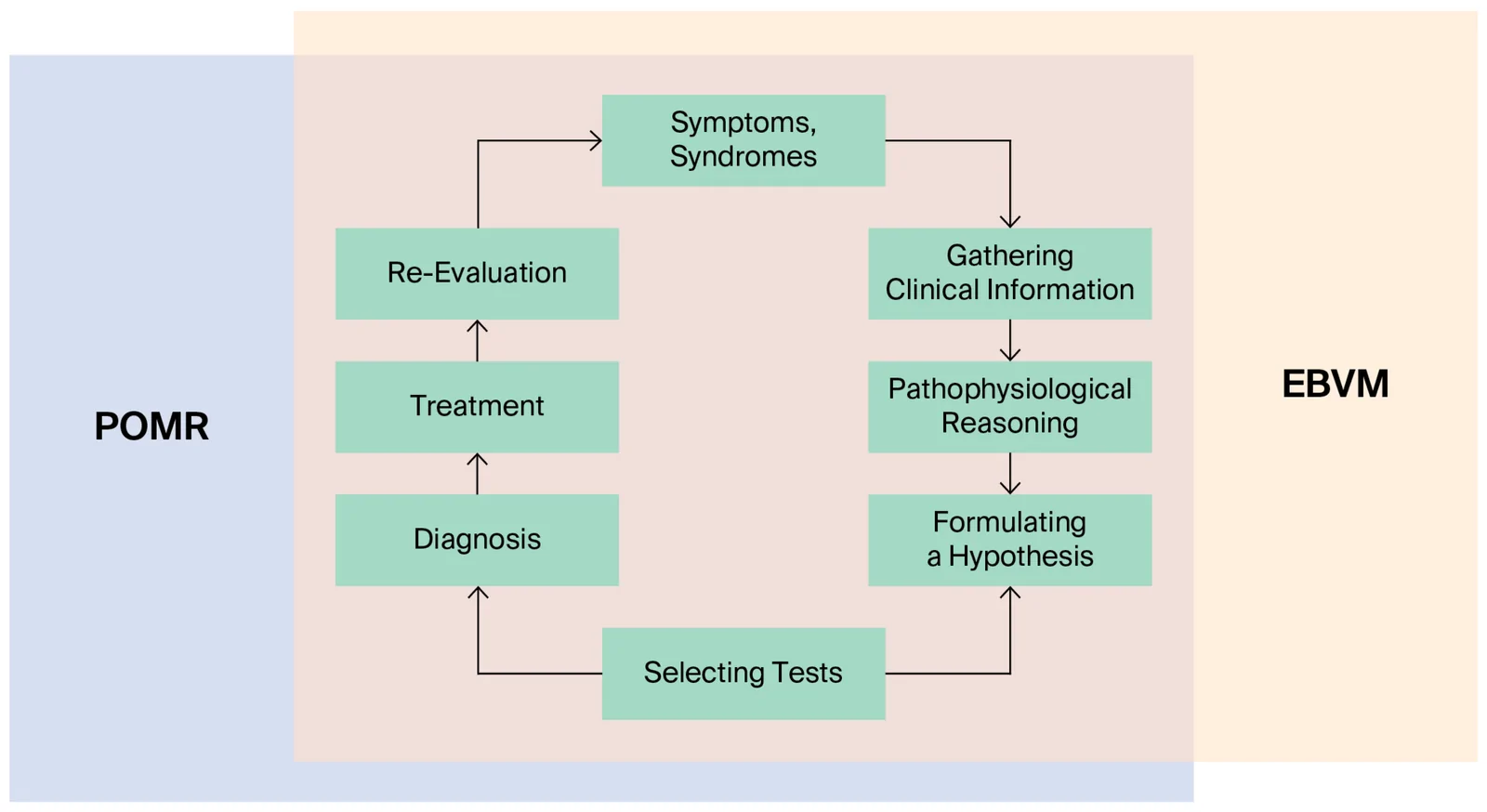
Clinical reasoning cycle of diagnosis and treatment in veterinary practice. POMR: problem-oriented medical record, EBVM: evidence-based veterinary medicine.

Clinical diagnosis and treatment are therefore best understood as an iterative cycle of clinical reasoning (Figure 2). Within this cycle, clinical information organized through the POMR is integrated with scientific evidence identified and appraised through EBVM and interpreted through pathophysiological reasoning to support patient-specific clinical decision-making.

The cognitive mechanisms underlying pathophysiological reasoning are discussed in the following section.

## 4. Theoretical Foundations

Clinical decision-making is grounded in systematized knowledge, professional and ethical norms, and the skills acquired through clinical training and experience. In actual clinical practice, however, decision-making is influenced by multiple interacting factors, including the patient’s condition, the owner’s values and preferences, the veterinarian’s experience and expertise, the human and material resources available within the veterinary facility, and time constraints. Consequently, clinical decision-making in individual cases is rarely explained adequately by a single theory. Instead, multiple theories of reasoning and cognition may contribute to complementary and context-dependent decision-making, according to the characteristics of the case and the clinical situation.

The central focus of this review is pathophysiological reasoning, which is considered within the broader and multifaceted processes of clinical reasoning and decision-making. The theoretical foundation of this review draws on concepts from several academic domains, including logic, cognitive science, decision-making research, clinical education, and the study of expertise. Although these theories are interrelated and may exhibit conceptual overlap, their theoretical foundations, explanatory targets, and intended applications are not necessarily identical.

Accordingly, this review focuses on the cognitive and reasoning processes addressed by each theoretical framework, as well as their relevance to clinical reasoning. Theoretical perspectives related to clinical reasoning encompass several distinct roles: providing the historical and normative foundations of reasoning; providing theoretical foundations for understanding human cognition; explaining the cognitive processes involved in clinical reasoning and decision-making; and informing the education and development of clinical reasoning expertise. On this basis, this section classifies major theories related to clinical reasoning into four categories according to their primary theoretical role and their contribution to understanding, performing, and developing clinical reasoning:

1. Historical and Normative Theories of Reasoning;
2. Foundational Theories of Modern Cognitive Science;
3. Theories of Clinical Reasoning and Decision-Making;
4. Theories of Clinical Education and Expertise.

This classification is not intended to divide the theories into mutually exclusive categories. Some concepts may be relevant to more than one category. Such overlap does not indicate theoretical contradiction; rather, it reflects the fact that a given theory may serve different explanatory purposes depending on the context. In this review, the theories are organized primarily according to their historical development and principal roles, and each category is presented as an overview of a particular dimension of clinical reasoning.

In actual clinical practice, multiple inferential and cognitive processes interact according to the patient’s condition, the clinical setting, and the purpose of decision-making. The theories presented here should therefore not be interpreted as prescriptive models that uniformly determine clinical judgment. Instead, they provide complementary conceptual frameworks for understanding different aspects of clinical reasoning. The final clinical decision and professional responsibility for each individual case remain with the veterinarian, who integrates the available clinical information, scientific evidence, patient circumstances, and clinical context.

Table 1 provides an overview of the principal theoretical frameworks considered relevant to clinical reasoning. The following sections describe the theoretical foundations and clinical relevance of each category.

### 4.1. Category 1: Historical and Normative Theories of Reasoning

Although the acquisition and interpretation of patient information are fundamental aspects of clinical practice, clinical decision-making also requires the selection of appropriate diagnostic and therapeutic actions. Evidence-based medicine (EBM) provides a framework in which the best available external evidence is integrated with individual clinical expertise and the circumstances and values of individual patients. Sackett et al. defined EBM as the conscientious, explicit, and judicious use of current best evidence in making decisions about the care of individual patients [61]. This approach followed the earlier recognition that clinical practice should be informed by systematically obtained research evidence rather than relying exclusively on traditional clinical authority [62]. In veterinary medicine, the corresponding concept of evidence-based veterinary medicine (EBVM) further emphasizes the integration of relevant scientific evidence with clinical expertise, patient circumstances, and the circumstances and values of the owner or carer.

The logical structure of clinical reasoning can be examined through complementary forms of inference, including deduction, induction, and abduction. These forms of inference provide different perspectives on how general knowledge and empirical observations may contribute to patient-specific clinical judgments [25,26,27]. Deductive reasoning applies established principles or propositions to clinical situations; inductive reasoning derives generalizations or probabilistic conclusions from observations and empirical data; and abductive reasoning generates plausible explanatory hypotheses that account for observed clinical findings.

These forms of inference should not be regarded as sequential or independent stages of EBM, as clinical reasoning may involve their dynamic interaction. Empirical observations can support inductive generalization, established knowledge can support the deductive evaluation of clinical hypotheses, and abductive reasoning can facilitate the generation of explanatory hypotheses for individual patients. Recent philosophical work has emphasized the complementary roles of deduction, induction, and abduction in medical reasoning, particularly the role of abduction in generating explanatory hypotheses in complex or atypical clinical situations [27].

This perspective also clarifies the relationship between EBM and logical reasoning. EBM should not be regarded as a formal decision-making system in which evidence automatically determines the appropriate clinical action. Instead, evidence must be interpreted in relation to individual patient characteristics, clinical expertise, patient and owner circumstances, and the practical context of care. Thus, while formal logic provides a normative framework for evaluating the validity of inferences, clinical decision-making extends beyond formal deduction and involves inductive and abductive reasoning as well as clinical judgment.

Within the theoretical framework adopted in this review, logic is positioned as a normative and epistemological foundation of clinical reasoning rather than as a comprehensive model of clinical decision-making. Its role is to provide principles for evaluating how conclusions follow from premises, how empirical observations support generalizations, and how explanatory hypotheses can be generated from clinical findings. Because these principles have historically contributed to philosophical accounts of scientific and clinical reasoning, logic is classified here within Historical and Normative Theories of Reasoning.

### 4.2. Category 2: Foundational Theories of Modern Cognitive Science

Advances in cognitive science shifted attention from questions concerning what constitutes valid reasoning to questions concerning how individuals process information, construct internal representations, and generate and revise interpretations. The development of EBM increased the emphasis on literature searching, critical appraisal, and the use of clinical guidelines, while the evolution of problem-oriented medical records (POMRs) and electronic medical records (EMRs) facilitated the systematic documentation, retrieval, and integration of clinical information. As clinical information and scientific evidence became increasingly accessible, greater attention was directed toward understanding how clinicians interpret clinical information and arrive at diagnostic judgments. Clinical diagnosis is an intellectual process involving information processing, inference, reasoning, and decision-making. Research in psychology and cognitive science has consequently contributed to several foundational frameworks relevant to clinical reasoning, including predictive processing, the Bayesian brain hypothesis, information processing approaches, schema theory, and mental model theory.

The following sections provide a concise overview of these frameworks and their relevance to clinical reasoning.

#### 4.2.1. Predictive Processing

Predictive processing theory proposes that perception is not a purely passive process driven by incoming sensory information. Instead, the brain continuously generates predictions based on internal models and compares these predictions with incoming sensory signals, with prediction errors contributing to the updating of internal representations [28,29,30]. Sensory information is therefore interpreted in the context of prior expectations and internal models rather than independently of them.

From this perspective, clinical reasoning can be conceptualized as involving continuously updated expectations regarding underlying pathophysiological states. Clinical history, physical examination findings, laboratory results, and imaging findings provide new information that may be consistent or inconsistent with the clinician’s current expectations, thereby contributing to the revision of diagnostic hypotheses.

#### 4.2.2. Bayesian Brain Hypothesis

The Bayesian brain hypothesis provides a theoretical framework in which the brain integrates prior knowledge with sensory information to perform probabilistic inference under conditions of uncertainty [31,32]. Rather than identifying a single interpretation independently of prior information, Bayesian approaches conceptualize perception and cognition as the estimation of the relative plausibility of competing hypotheses.

Applied to clinical reasoning, this framework provides a conceptual basis for understanding how clinicians integrate prior knowledge and contextual information with incoming clinical evidence under uncertainty. Initial diagnostic hypotheses may be influenced by prior experience, disease prevalence, and patient-specific circumstances, and may subsequently be revised as additional clinical findings, laboratory results, and imaging findings become available. This iterative updating provides a theoretical explanation for the revision of diagnostic hypotheses as new evidence is acquired.

#### 4.2.3. Information Processing Approach

Information processing approaches conceptualize human cognition as a set of interacting processes through which sensory information is encoded, attended to, temporarily maintained and manipulated in working memory, integrated with information retrieved from long-term memory, and ultimately transformed into cognitive judgments or behavioral responses [33,34,35,36]. Within this framework, reasoning involves multiple interacting processes, including perception, attention, working memory, long-term memory, retrieval, information integration, and response selection.

This framework therefore provides a conceptual basis for understanding how clinicians acquire clinical information, selectively attend to relevant findings, retrieve prior knowledge, integrate new information with existing knowledge, and generate diagnostic and therapeutic judgments.

#### 4.2.4. Schema Theory

Schema theory provides a framework for understanding how knowledge is organized into structured cognitive representations and how previously acquired knowledge influences the interpretation of new information [37]. Schemas represent organized knowledge structures involving relationships among concepts, experiences, and expectations. When new information is encountered, relevant schemas may be activated and provide a framework for interpreting and integrating that information with existing knowledge.

#### 4.2.5. Mental Model Theory

Mental model theory proposes that individuals construct internal representations of situations and use these representations to reason about possible states of the world rather than relying exclusively on abstract logical rules [38,39]. In clinical reasoning, this perspective can be applied to the construction of internal representations of a patient’s clinical and pathophysiological state. Such representations may help clinicians reason about causal relationships, disease progression, and possible responses to diagnostic or therapeutic interventions.

### 4.3. Category 3: Theories of Clinical Reasoning and Decision-Making

EBM provides methods for identifying and applying relevant research evidence to clinical problems but it does not by itself provide a comprehensive account of how clinicians recognize clinical problems, formulate diagnostic hypotheses, interpret incomplete information, or make decisions under uncertainty. Cognitive and decision-making theories have therefore been developed to explain different aspects of clinical reasoning and decision-making. Representative frameworks include dual-process theory, illness script theory, the hypothetico-deductive model, forward reasoning, naturalistic decision-making, and situated cognition.

#### 4.3.1. Dual-Process Theory

Dual-process theories propose that human reasoning involves qualitatively different modes of cognitive processing. Type 1 processing is generally rapid, relatively automatic, and associated with intuitive judgments, whereas Type 2 processing is slower, more effortful, and more deliberative [40,41]. In clinical reasoning, these processes are often described as System 1 and System 2, respectively. The framework provides a conceptual basis for understanding how experienced clinicians may rapidly recognize familiar clinical patterns while retaining the capacity to engage in more deliberate analytical reasoning when cases are atypical, ambiguous, or diagnostically challenging.

This framework is particularly relevant to veterinary medicine. Canfield et al. (2016) [42] described the complementary use of intuitive and analytical reasoning in feline clinical practice and emphasized the potential value of combining pattern recognition with analytical, problem-oriented reasoning.

#### 4.3.2. Illness Script Theory

Illness script theory proposes that clinical knowledge is organized into structured representations of diseases and clinical conditions, referred to as illness scripts. These scripts integrate enabling conditions, pathophysiological mechanisms or faults, and their clinical consequences [23,43,44]. Through clinical experience, illness scripts become increasingly elaborated and may facilitate the rapid interpretation of clinical findings.

During clinical reasoning, relevant illness scripts may be activated by patient characteristics and clinical findings. Script activation can facilitate rapid interpretation and may guide the selection of subsequent diagnostic actions. When several possible scripts are activated, clinicians may compare alternative explanations and use additional information to refine or revise their diagnostic hypotheses.

#### 4.3.3. Hypothetico-Deductive Model

The hypothetico-deductive model describes a cyclical process in which clinicians formulate preliminary diagnostic hypotheses from initial clinical information and then selectively collect additional information to test, refine, or reject those hypotheses [45,46]. These hypotheses can subsequently guide medical history-taking, physical examination, and diagnostic testing. Diagnostic reasoning is therefore an active process in which information acquisition and hypothesis evaluation interact continuously rather than a passive accumulation of clinical information.

The hypothetico-deductive model emphasizes the early generation of diagnostic hypotheses and the strategic collection of information relevant to evaluating those hypotheses. It provides a conceptual framework for understanding how clinicians determine which additional information should be sought and how newly acquired information may lead to the modification or rejection of an initial diagnostic interpretation.

#### 4.3.4. Forward Reasoning

Forward reasoning is a data-driven reasoning strategy that proceeds from observed clinical findings through established causal and pathophysiological knowledge toward explanatory hypotheses and diagnostic conclusions [42,47,48]. In contrast to reasoning that begins with a diagnostic hypothesis and works backward to determine whether the available findings support it, forward reasoning begins with the findings themselves and follows relevant physiological, pathological, and pathophysiological relationships toward possible explanations.

This approach emphasizes the importance of understanding anatomy, physiology, pathology, and pathophysiology as interconnected causal systems rather than merely memorizing disease-specific features. Patel and Groen (1986) [47] reported that accurate diagnostic performance in a specific clinical reasoning task was characterized by knowledge-driven forward reasoning. However, subsequent research has indicated that the relationship among reasoning strategy, expertise, and task characteristics is complex. Forward reasoning should therefore be regarded as an important reasoning strategy rather than as a universal marker of expertise.

In the framework proposed in this review, forward reasoning provides an important theoretical basis for pathophysiological reasoning because it enables clinicians to interpret clinical findings through underlying biological mechanisms and causal relationships. Pathophysiological reasoning, however, extends beyond forward reasoning alone by integrating clinical findings, contextual information, diagnostic evidence, and clinical judgment in the formulation of patient-specific explanations and decisions.

#### 4.3.5. Naturalistic Decision-Making

Naturalistic decision-making theory examines how experienced decision-makers make judgments and select courses of action in complex, dynamic, and uncertain real-world environments [49,50]. Rather than assuming that decision-makers systematically compare all possible alternatives, naturalistic decision-making emphasizes the roles of experience, situation recognition, pattern recognition, and mental simulation.

Within recognition-primed decision-making approaches, experienced decision-makers may recognize a plausible course of action based on the current situation and mentally simulate its potential consequences rather than systematically comparing multiple alternatives. If the proposed course of action appears plausible, it may be implemented; if the simulation reveals problems, the decision-maker may modify or replace the proposed action. This framework provides a useful explanation for how experienced clinicians may arrive at decisions without explicitly evaluating numerous alternatives.

#### 4.3.6. Situated Cognition

Situated cognition views cognition and knowledge as processes that are shaped by the activities, environments, and social contexts in which they are used rather than as phenomena occurring exclusively within an individual’s mind [51,52,53]. Applied to clinical reasoning, this perspective emphasizes that reasoning emerges through interactions among clinicians, patients, owners, members of the healthcare team, diagnostic technologies, and other material and social resources.

Situated cognition therefore expands the scope of clinical reasoning beyond individual cognitive processes and highlights the influence of contextual, environmental, social, and material factors on diagnostic and clinical decision-making.

### 4.4. Category 4: Theories of Clinical Education and Expertise

Theories of clinical reasoning focus primarily on how clinicians reason and make decisions. In contrast, educational and expertise-related frameworks address how these capabilities are acquired, monitored, refined, and developed over time. Metacognition, reflective practice, and the Clinical Reasoning Cycle provide complementary educational frameworks for fostering, assessing, and refining clinical reasoning throughout professional development.

#### 4.4.1. Metacognition

Metacognition refers to the awareness, monitoring, and regulation of one’s own cognitive processes [54,55,56]. Rather than replacing intuitive or analytical reasoning, metacognition operates at a higher level by allowing clinicians to monitor their ongoing reasoning, assess the adequacy of their judgments, and initiate re-evaluation when uncertainty, inconsistency, or potential error is recognized. Its potential contributions to clinical reasoning include monitoring diagnostic judgments, recognizing possible cognitive biases, calibrating confidence, managing uncertainty, supporting self-regulated learning, and promoting adaptive expertise.

#### 4.4.2. Reflective Practice

Reflective practice refers to a deliberate process in which practitioners critically examine their experiences, actions, assumptions, and reasoning processes to derive insights that may improve future practice and performance [57,58,59]. In clinical reasoning, reflective practice may involve revisiting diagnostic judgments, examining assumptions underlying initial hypotheses, and reconsidering alternative explanations when clinical findings are inconsistent with the initial interpretation.

Reflective practice may contribute to professional development by promoting awareness of one’s reasoning processes, learning from diagnostic successes and failures, integrating theoretical knowledge with clinical experience, and adapting reasoning when clinical situations deviate from familiar patterns. Deliberate reflection may also contribute to improved diagnostic accuracy in selected clinical reasoning contexts.

#### 4.4.3. Clinical Reasoning Cycle

The Clinical Reasoning Cycle (CRC) is an educational model that conceptualizes clinical reasoning as a dynamic and iterative process comprising eight interrelated stages: (1) considering the patient situation and/or client, (2) collecting cues and information, (3) processing information, (4) identifying problems and issues, (5) establishing goals, (6) taking action, (7) evaluating outcomes, and (8) reflecting on the process and new learning [60]. Although presented as eight stages, the CRC is not intended to represent a strictly linear process. Clinicians may move back and forth between stages, revisit earlier stages, or engage in multiple stages concurrently as the clinical situation evolves.

The CRC has also been adapted to veterinary medical education. Carr et al. (2024) [8] and Ferlini Agne et al. (2024) [9] demonstrated its potential use as an explicit and teachable framework for guiding veterinary learners through data collection, analysis, clinical action, evaluation, and reflection. The CRC should therefore be regarded primarily as an educational framework rather than as a comprehensive cognitive theory of clinical reasoning.

### 4.5. Integration of the Theoretical Perspectives and Connection to Pathophysiological Reasoning

The diversification of clinical reasoning theories over recent decades does not necessarily imply that these theories represent competing paradigms. Rather, they address different explanatory questions concerning how clinicians acquire, organize, interpret, apply, monitor, and refine clinical knowledge. Their partial conceptual overlap is therefore expected, because the same clinical event can be considered from multiple cognitive and practical perspectives.

The present framework integrates these perspectives by assigning them different conceptual contributions to a common clinical process. Historical and normative theories provide principles for evaluating inference and the relationship between evidence and conclusions. Foundational theories of cognitive science describe how information is predicted, processed, represented, and updated. Theories of clinical reasoning and decision-making explain how clinicians generate, test, and refine hypotheses and select actions under uncertainty and contextual constraints. Theories of clinical education and expertise address how these processes are monitored, reflected upon, taught, and developed over time. These perspectives therefore contribute different explanatory components rather than competing definitions of the same process.

Within this synthesis, pathophysiological interpretation provides the central clinical link between these theoretical contributions. Clinical information is first acquired and organized; it is then interpreted in relation to anatomy, physiology, pathology, and disease mechanisms; the resulting pathophysiological model is used to assess severity and prognosis; and this interpretation contributes to patient-specific clinical decision-making. Pathophysiology-centered clinical reasoning (PCCR) is derived from this integrative relationship rather than from any single existing theory. It therefore does not replace forward reasoning, illness scripts, POMR, EBVM, or the Clinical Reasoning Cycle. Forward reasoning, for example, provides an important mechanism for moving from findings toward causal explanations, whereas illness scripts organize disease-related knowledge and the Clinical Reasoning Cycle provides an educational structure for iterative clinical reasoning. POMR organizes clinical information, and EBVM supports the identification and application of external scientific evidence. PCCR instead provides a common pathophysiology-centered clinical organization through which these complementary processes can be connected in an individual case.

The four categories described above therefore represent different dimensions of clinical reasoning, whereas pathophysiological reasoning represents an integrative clinical process through which these dimensions may be brought together in patient-specific reasoning.

Accordingly, PCCR should be understood as a clinical integrative framework, not as a new cognitive theory or a claim that clinicians consciously apply all 15 theoretical frameworks during every encounter. Its proposed added value is to make explicit the pathophysiological interpretation that connects diverse forms of clinical information and theoretical reasoning processes with assessment of severity, prognosis, and subsequent clinical decision-making.

## 5. Clinical Reasoning in Veterinary Ophthalmology

A systematic approach to ophthalmic examination was already documented in veterinary ophthalmology by the 1970s and became increasingly consolidated in major ophthalmology texts published around the late 1970s and early 1980s. An organized approach to ophthalmic examination was described in the second edition of Glenn A. Severin’s *Veterinary Ophthalmology Notes* [63], which included dedicated sections on the examination of the eye, ophthalmoscopy, and the evaluation of blindness. This was followed by several influential veterinary ophthalmology textbooks, including Blogg’s *The Eye in Veterinary Practice: Extraocular Disease* [64], Gelatt’s *Textbook of Veterinary Ophthalmology* [65], and Slatter’s *Fundamentals of Veterinary Ophthalmology* [66]. These publications contributed to the increasing organization of ophthalmic examination and diagnostic procedures as integral components of veterinary ophthalmic practice.

Over subsequent decades, ophthalmic examination and diagnostic procedures became increasingly standardized and were incorporated into practical approaches to the evaluation of dogs and cats. Later ophthalmology textbooks and clinical publications further refined the sequence, interpretation, and application of individual examination techniques. Contemporary references describe the complete ophthalmic examination as a systematic process that integrates history-taking, external and internal ocular examination, neuro-ophthalmic assessment, the assessment of tear production and corneal integrity, tonometry, evaluation of the nasolacrimal system, ophthalmoscopy, and the assessment of vision [67,68,69].

Through this historical development, ophthalmic examination evolved from the use of individual diagnostic techniques toward an increasingly integrated clinical examination framework. The contemporary concept of the complete ophthalmic examination can therefore be understood as the result of the progressive integration and standardization of multiple diagnostic components.

From the perspective of clinical reasoning, the complete ophthalmic examination provides a structured framework for acquiring and organizing clinical information. The findings obtained through the examination provide the evidentiary basis for identifying clinically relevant features, constructing problem representations, formulating and evaluating differential diagnoses, and making diagnostic and therapeutic decisions. In veterinary clinical reasoning, these processes are closely linked to systematic data collection and analysis, problem representation, differential diagnosis, management planning, and subsequent reflection and reassessment [9].

The complete ophthalmic examination is therefore not merely a collection of diagnostic techniques but a structured process for integrating findings from multiple anatomical and functional domains of the visual system. By relating clinical signs to anatomical abnormalities and associated functional disturbances, the examination provides the clinical information required to infer underlying pathophysiological processes. In this sense, the complete ophthalmic examination represents a foundational process of information acquisition, organization, and problem representation within clinical reasoning.

This perspective is particularly relevant to veterinary ophthalmology because ocular findings may represent abnormalities at different anatomical and functional levels, ranging from the ocular surface and anterior segment to the retina, optic nerve, and central visual pathways. Consequently, the interpretation of ophthalmic findings requires clinicians to move beyond the identification of isolated abnormalities and consider how individual findings can be integrated into a coherent pathophysiological explanation.

**Figure 3:**
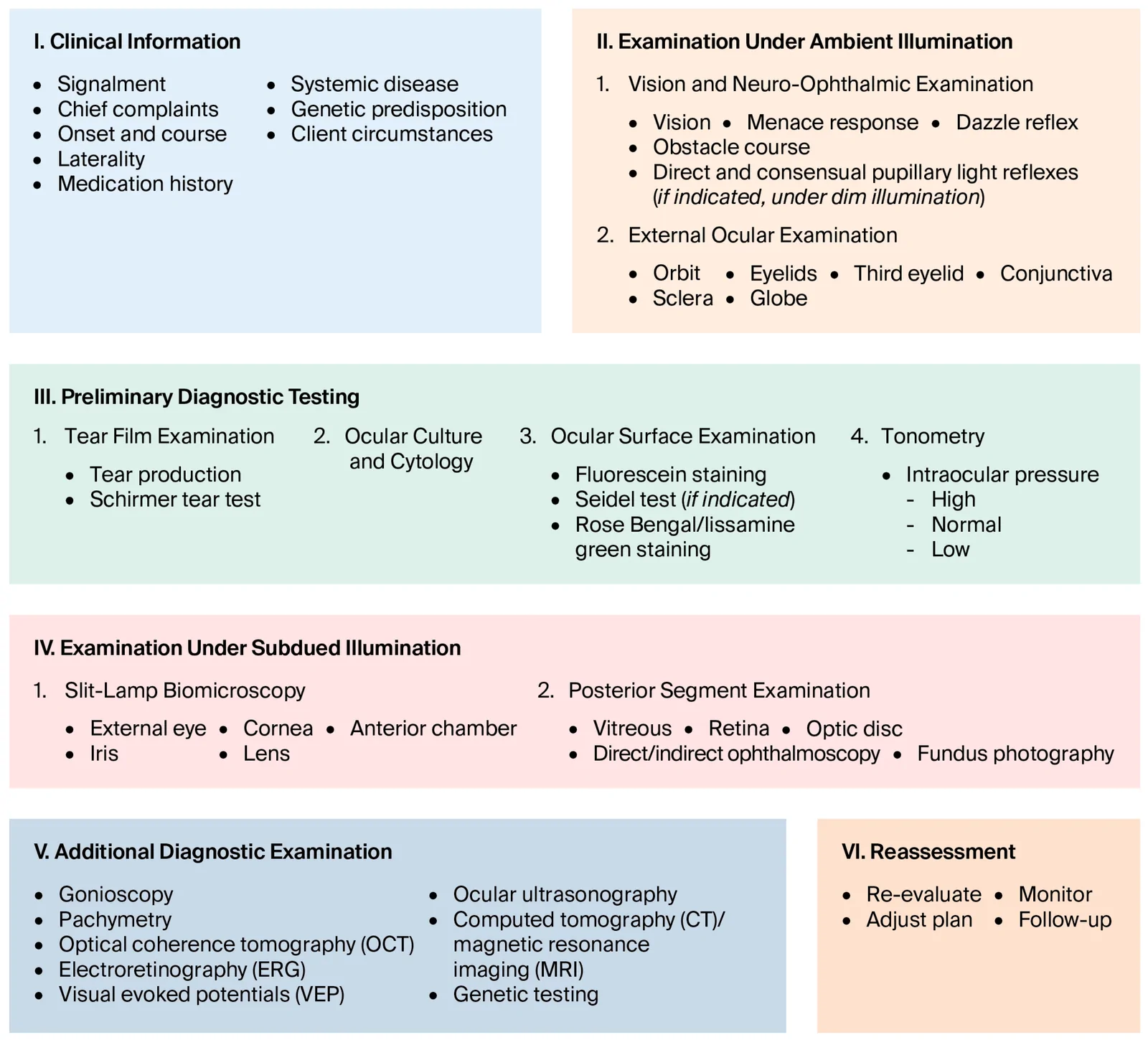
A comprehensive guideline for ophthalmic examination in canine and feline clinical practice.

Figure 3 presents a comprehensive framework for the complete ophthalmic examination in veterinary ophthalmology. The framework is intended not only to illustrate the sequence of examination procedures but also to emphasize how information obtained from each component contributes to the interpretation of ocular findings, formulation of problem representations, and subsequent clinical decision-making.

The application of this framework to specific ophthalmic disorders is illustrated in the following section through representative clinical scenarios involving glaucoma, a descemetocele with aqueous humor leakage, and traumatic proptosis.

## 6. Representative Clinical Scenarios

A comprehensive ophthalmic examination provides the foundation for information acquisition and problem representation in clinical reasoning. However, how the clinical information obtained through this examination is interpreted and translated into clinical decisions—including diagnosis, pathophysiological understanding, prognosis assessment, and treatment planning—depends on the theoretical and cognitive processes involved. The three clinical scenarios were selected because they represent distinct pathophysiological and decision-making contexts commonly encountered in veterinary ophthalmic practice: a pressure-related optic neuropathy requiring etiological interpretation (glaucoma), a structural corneal emergency requiring assessment of tissue integrity and urgency (descemetocele with aqueous humor leakage), and an acute traumatic condition requiring assessment of multiple interacting ocular and neurological consequences (traumatic proptosis). Together, these cases provide contrasting contexts in which the same overarching pathophysiology-centered sequence can be illustrated.

In this section, established clinical reasoning theories are applied to three representative clinical scenarios to illustrate different ways in which clinical information obtained at the initial outpatient visit can be interpreted from a pathophysiological perspective. The scenarios encompass glaucoma in a Shiba Inu, descemetocele with aqueous humor leakage in a Japanese Domestic Cat, and traumatic proptosis in a Chihuahua. The cases were selected for conceptual illustration rather than empirical validation of PCCR or comparison of the relative validity of the individual theories.

In this review, PCCR is not presented as a distinct cognitive theory. Rather, it is proposed as an integrative clinical reasoning framework that emphasizes pathophysiological interpretation as a central link between clinical information, assessment of severity and prognosis, and clinical decision-making. The framework can be represented as: clinical information → pathophysiological interpretation → severity and prognosis assessment → clinical decision-making. The theoretical perspectives reviewed in this article provide different ways of supporting or explaining the processes occurring within this sequence; PCCR provides the common clinical organization in which they can be considered together.

Scenario 1 focuses on glaucoma and illustrates how abnormalities in aqueous humor dynamics can be interpreted to construct a pathophysiological model of functional impairment and optic nerve damage. Scenario 2 uses a descemetocele with aqueous humor leakage to demonstrate how the structural disruption of the cornea can be interpreted in terms of tissue integrity, aqueous humor leakage, and the urgency of intervention. Scenario 3 uses traumatic proptosis to illustrate how an initial anatomical injury can trigger a sequence of pathophysiological changes, including vascular compromise, neurological dysfunction, and corneal exposure and drying. Together, these three scenarios demonstrate that the application of different clinical reasoning theories varies according to the underlying pathophysiology, while sharing a common integrative framework centered on PCCR.

### 6.1. Scenario 1: Glaucoma in a Shiba Inu

#### 6.1.1. Case

A 10-year-and-2-month-old female Shiba Inu weighing 13.9 kg was presented to our clinic. Approximately two months before presentation, the dog developed photophobia and blepharospasm in the left eye following a grooming session. She was subsequently diagnosed with glaucoma and treated with 0.5% timolol and 0.05% latanoprost ophthalmic solutions twice daily. She was referred to our clinic because epiphora occurred immediately after topical medication administration.

**Figure 4:**
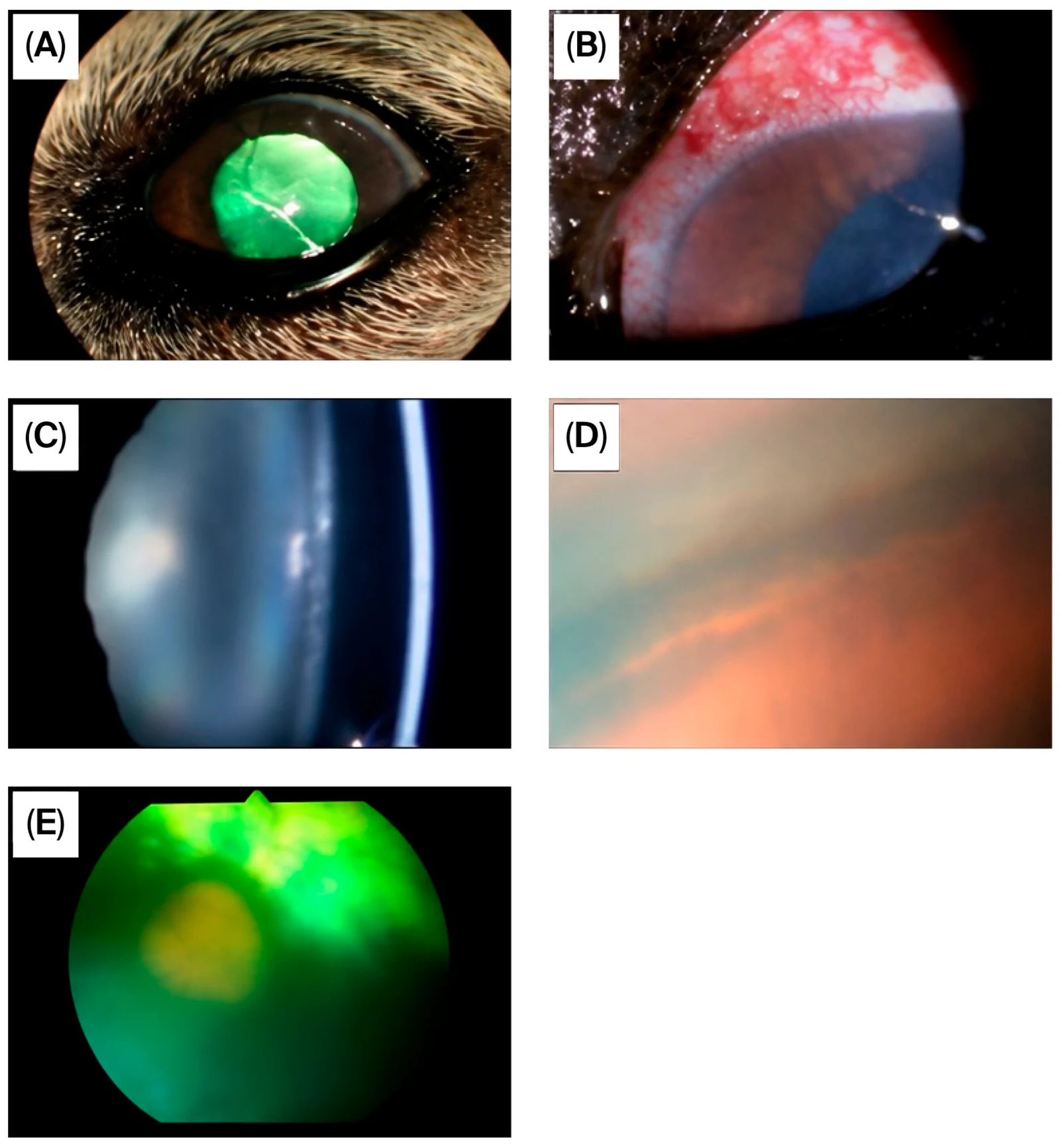
The left eye of a Shiba Inu with glaucoma, as described in Scenario 1. (**A**) External appearance; (**B**) episcleral vascular congestion; (**C**) slit-lamp image of the anterior segment; (**D**) anterior chamber angle image showing angle closure; (**E**) optic disc cupping, disappearance of retinal vessels, and increased tapetal reflectivity.

At presentation, the left eye showed dilation of the superior scleral vessels, conjunctival hyperemia, and ocular pain. Intraocular pressure (IOP) was persistently elevated despite treatment, with measurements of 29, 26, and 25 mmHg (mean, 27 mmHg). The Schirmer tear test (STT) value was 27 mm/min. A mild direct pupillary light reflex was present, and atrophy was observed at the pupillary margin. The iridocorneal angle was closed. A Y-shaped suture opacity was observed in the lens but there was no evidence of lens displacement. Funduscopic examination revealed retinal vascular attenuation and optic disk cupping (Figure 4). Shiba Inus have the highest incidence of glaucoma in Japan, which is attributed to their high rate of abnormal or narrow iridocorneal angles (ICAs), observed in both glaucomatous and nonglaucomatous eyes compared with other breeds [70,71]. Since abnormal ICAs increase the risk of glaucoma, it is presumed that the glaucoma that developed spontaneously in the Shiba Inu is primary glaucoma.

Based on these findings, a diagnosis of primary angle-closure glaucoma in the left eye was established.

#### 6.1.2. Examples of the Application of Clinical Reasoning Theories

Table 2 presents concise examples of how different clinical reasoning theories can be used to interpret the clinical information obtained in this case. Predictive processing, the Bayesian brain, the information processing approach, and schema theory provide perspectives on how clinical information may be predicted, selected, organized, and integrated. Mental model theory helps explain the construction of a causal representation linking the observed findings to the underlying pathophysiology. Dual-process theory, illness script theory, the hypothetico-deductive model, and forward reasoning provide complementary perspectives on diagnostic hypothesis generation and evaluation. Naturalistic decision-making and situated cognition emphasize the relationship between reasoning, clinical circumstances, and decision-making, whereas metacognition, reflective practice, and the clinical reasoning cycle emphasize monitoring, evaluation, and reflection.

This case illustrates an integrated clinical reasoning process in which initial information, including breed and age, is incorporated into the clinical context; clinical information is collected and interpreted; a pathophysiological model is constructed; the hypothesis of primary angle-closure glaucoma is formulated and evaluated against alternative explanations; and clinical management is determined. The pathophysiological model may subsequently be reevaluated and updated as additional information becomes available.

The integration of multiple theoretical frameworks within a single case allows the same clinical information to be examined from complementary perspectives, thereby facilitating a multifaceted understanding of clinical reasoning.

### 6.2. Scenario 2: Descemetocele with Aqueous Humor Leakage in a Japanese Domestic Cat

#### 6.2.1. Case

A 13-year-and-8-month-old neutered male Japanese domestic cat weighing 6.46 kg was presented to our clinic. Ocular discharge and persistent eyelid closure of the right eye had been observed for approximately two months, during which the cat had received treatment. Three weeks before presentation, the cat developed ocular pain and was examined at a referring hospital, where treatment with 0.3% lomefloxacin, 3% N-acetylcysteine, and 0.1% sodium hyaluronate ophthalmic solutions, administered three times daily, was initiated. The cat was subsequently referred to our clinic because of suspected corneal perforation.

**Figure 5:**
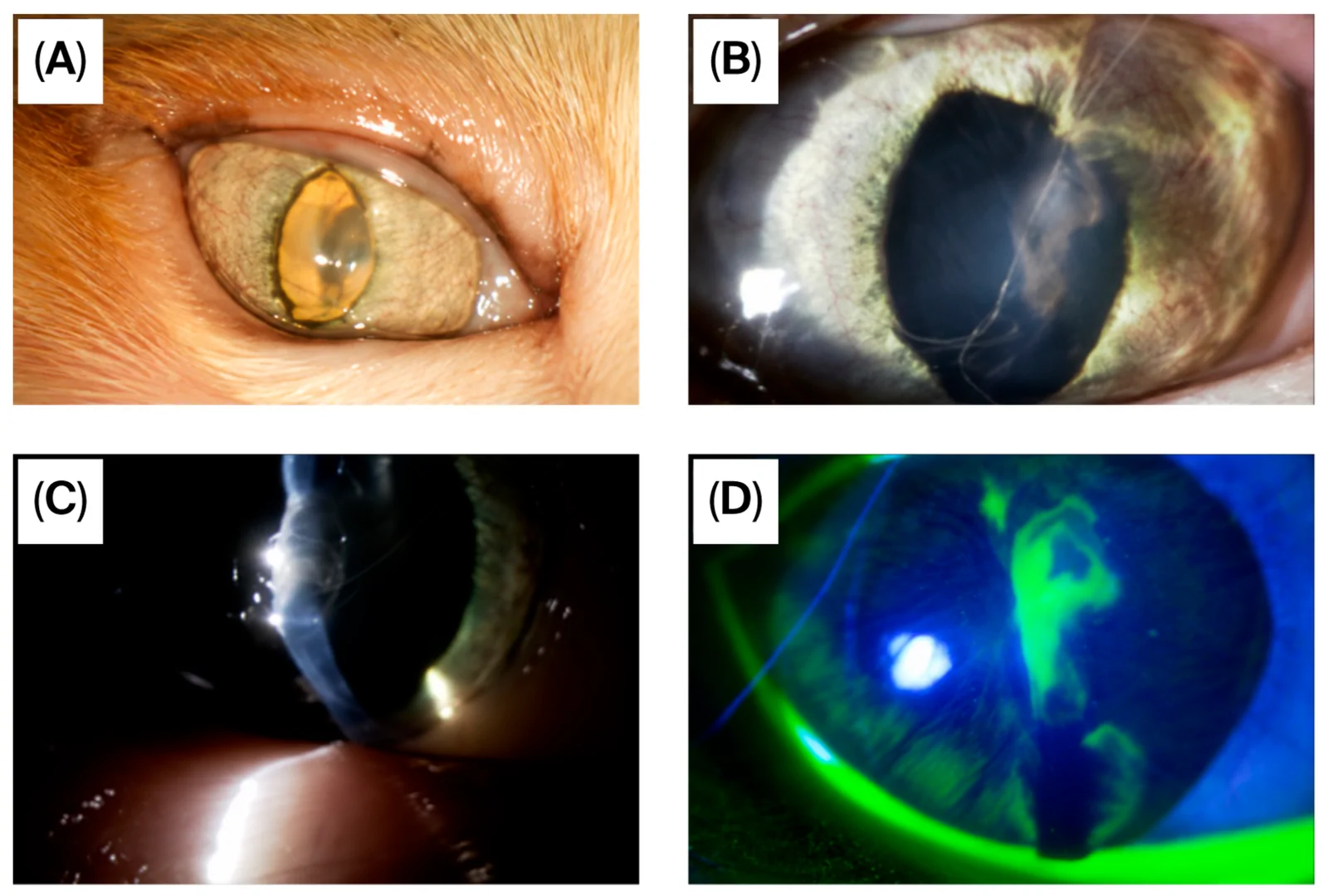
The right eye of a Japanese domestic cat with a descemetocele and aqueous humor leakage, as described in Scenario 2. (**A**) External appearance of the descemetocele with aqueous humor leakage; (**B**) anterior segment image immediately after aqueous humor leakage was observed; (**C**) slit-lamp image immediately after aqueous humor leakage was observed; (**D**) external appearance demonstrating a positive Seidel test.

A descemetocele measuring approximately 2 × 4 mm was observed in the central region of the right cornea during the initial examination. The lesion was accompanied by ocular pain and ciliary hyperemia. The pupil showed a tendency toward miosis, whereas the menace response, dazzle reflex, and direct pupillary light reflex were present. The Seidel test was positive, confirming aqueous humor leakage (Figure 5). The STT value was 25 mm/min.

Based on these findings, a diagnosis of descemetocele with aqueous humor leakage in the right eye was established. The lesion represented extreme corneal thinning with disruption of the cornea to the level of Descemet’s membrane and confirmed aqueous humor leakage, consistent with corneal perforation.

#### 6.2.2. Examples of the Application of Clinical Reasoning Theories

Table 3 presents concise examples of how different clinical reasoning theories can be used to interpret the same clinical information. In this case, logic and normative reasoning, mental model theory, the hypothetico-deductive model, and the clinical reasoning cycle are particularly useful for clarifying the relationship between severe corneal structural disruption and aqueous humor leakage. Predictive processing, the Bayesian brain, the information processing approach, and schema theory provide complementary perspectives on how information concerning disease progression and structural integrity can be interpreted. These examples illustrate how the same clinical findings can be interpreted from different theoretical perspectives, progressing from morphological and functional findings to pathophysiological interpretation, assessment of urgency, and clinical decision-making.

### 6.3. Scenario 3: Traumatic Proptosis in a Chihuahua

#### 6.3.1. Case

A 1-year-old male Chihuahua weighing 2.3 kg was presented to our clinic. Two days before presentation, at approximately 6:00 PM, the right eye became proptotic following contact with a Shiba Inu at a park. The dog was immediately taken to a primary veterinary hospital, where 0.3% ofloxacin and 0.1% sodium hyaluronate ophthalmic solutions were administered hourly, together with 0.005% latanoprost ophthalmic solution.

**Figure 6:**
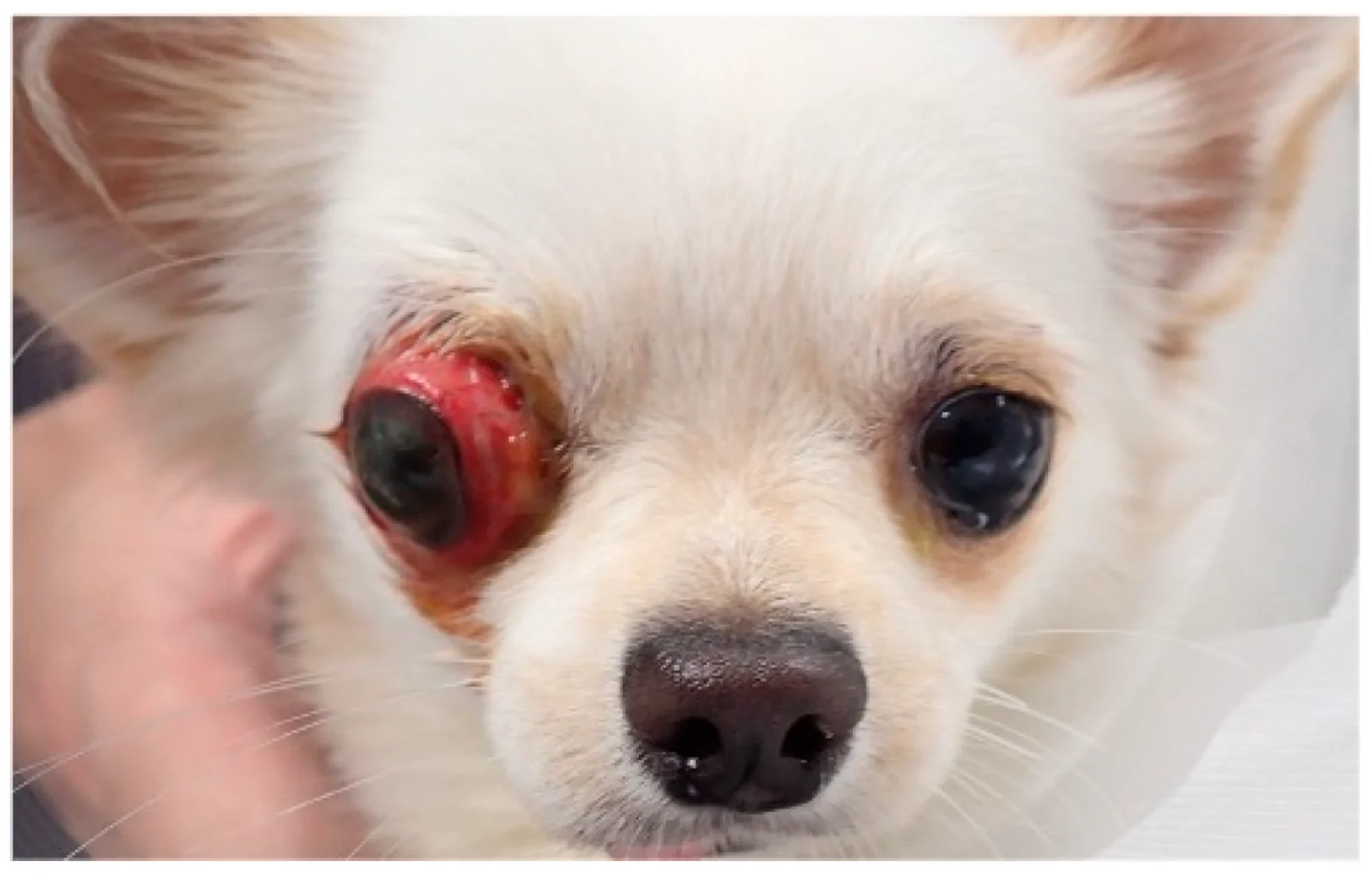
The external appearance of a Chihuahua with traumatic proptosis, as described in Scenario 3. Protrusion of the right globe with exposure of the equatorial region.

Initial examination revealed anterior displacement of the right globe beyond the palpebral fissure at the level of the equator (Figure 6). Severe eyelid swelling and edema were present. The eyelid margin was inverted, and subcutaneous hemorrhage was observed in the lateral eyelid skin. The globe was laterally displaced, and the corneal surface was dry. The conjunctiva was inflamed and edematous, with small lacerations along the lateral and medial sclera. The anterior chamber was clear, and the pupil was 2.5 mm in diameter and circular. Although a direct pupillary light reflex was absent, the menace response and dazzle reflex were present.

Based on these findings, a diagnosis of traumatic proptosis in the right eye was established.

#### 6.3.2. Examples of the Application of Clinical Reasoning Theories

Table 4 presents concise examples of how different clinical reasoning theories can be used to interpret the clinical information obtained in this case. The information processing approach provides a framework for understanding the sequential collection and integration of clinical information, whereas situated cognition emphasizes how patient- and practice-specific circumstances can influence clinical decision-making. Logic and normative reasoning, predictive processing, the Bayesian brain, mental model theory, and the hypothetico-deductive model, together with forward reasoning, provide complementary perspectives on pathophysiological interpretation and prediction of potential disease progression.

These theoretical perspectives demonstrate that clinical reasoning extends beyond identifying a disease name. Instead, it involves translating clinical information into a pathophysiological model that can be used to assess the severity of tissue injury, anticipate potential functional consequences, estimate the prognosis for vision and globe preservation, and determine appropriate clinical actions.

### 6.4. Synopsis of the Three Clinical Scenarios

The three clinical scenarios presented above illustrate different pathways from clinical information to pathophysiological interpretation and clinical decision-making. Scenario 1, glaucoma in a Shiba Inu, illustrates reasoning concerning the pathophysiology and etiology of disease, with particular emphasis on abnormalities in aqueous humor dynamics, persistent elevation of IOP, and optic nerve damage. Scenario 2, descemetocele with aqueous humor leakage in a Japanese domestic cat, illustrates reasoning from structural disruption of the cornea to aqueous humor leakage and the resulting clinical urgency. Scenario 3, traumatic proptosis in a Chihuahua, illustrates reasoning from trauma-induced anatomical disruption to tissue injury, potential vascular and neurological compromise, corneal exposure, and assessment of the possibility of preserving the globe and vision.

Across these scenarios, the PCCR provides a common integrative framework for connecting diverse forms of clinical information with pathophysiological interpretation and subsequent clinical decision-making. The cases demonstrate that clinical reasoning extends beyond identification of a disease name; rather, clinical information is interpreted in the context of anatomy, physiology, and pathophysiology, a pathophysiological model is constructed and refined, severity and prognosis are assessed, and clinical decisions are subsequently made.

## 7. Future Perspectives

The PCCR framework proposed in this review is a conceptual synthesis derived from the reviewed literature and clinical examples rather than an empirically validated cognitive theory or clinical prediction model. The three clinical scenarios were selected to illustrate its application in veterinary ophthalmology and were not intended to provide empirical validation. Future research should therefore evaluate whether the PCCR sequence can support clinical reasoning education, assessment, and practice across different veterinary disciplines. Such investigations could examine whether learners and clinicians can reliably identify and articulate pathophysiological links between clinical findings and clinical decisions, whether the framework improves the explicit teaching or evaluation of clinical reasoning, and whether its use is associated with clinically meaningful outcomes. Until such evidence is available, its broader applicability beyond veterinary ophthalmology should be considered a hypothesis rather than an established generalizable conclusion.

Clinical reasoning is increasingly addressed as an explicit educational competency, with recent reviews emphasizing structured curricula, deliberate reflection, case-based learning, and other approaches that support the development and assessment of reasoning skills [8,9]. Future research should determine whether the PCCR sequence can be integrated into veterinary education and whether learners can reliably articulate the pathophysiological links between clinical findings and clinical decisions.

The potential contribution of emerging technologies is also relevant. Recent veterinary literature highlights the growing use of artificial intelligence and language models in clinical practice and education, while emphasizing validation, transparency, clinician oversight, and critical evaluation of AI-generated information [72,73,74].

At the same time, the implementation of clinical reasoning is fundamentally dependent on the acquisition and interpretation of patient-specific clinical information. Consequently, advances in diagnostic technologies may substantially alter the nature of clinical reasoning and decision-making. Emerging technologies include artificial intelligence (AI), deep learning, computer-aided diagnostic approaches and automated image analysis, radiomics and quantitative imaging, three-dimensional imaging and reconstruction, ultrasonography and point-of-care ultrasound, multimodal imaging, and multimodal AI and large language models [72,73,74,75,76,77,78,79]. However, many of these technologies remain at an early stage of development and require rigorous validation before their widespread adoption in clinical practice. As demonstrated by the comprehensive ophthalmic examination, which has long served as a cornerstone of veterinary ophthalmology, the clinical value of new diagnostic approaches must ultimately be established through systematic evaluation by clinicians and researchers. Importantly, the emerging integration of AI into veterinary clinical decision-making also raises questions concerning explainability, professional judgment, potential biases, and the appropriate balance between technological assistance and clinician responsibility [80,81,82].

Another emerging area is the application of advances in animal cognitive science, particularly canine cognition, to veterinary clinical practice. Recent research has expanded our understanding of canine behavior, cognition-related responses, emotional states, and responses to handling and environmental stimuli [83,84,85,86,87]. From a clinical perspective, animal behavior should not be regarded merely as an obstacle to examination or treatment but as clinically relevant information reflecting patients’ responses to their environment, pain, fear, and other affective states [88,89,90,91,92]. Combining such cognitive and behavioral information with conventional clinical findings may facilitate more individualized approaches to patient handling, behavioral assessment, and welfare evaluation. This perspective may be particularly important when treatment tolerance, quality of life, and animal welfare are major considerations in clinical decision-making.

Furthermore, veterinary clinical decision-making has received increasing attention as an important area of research and clinical practice [93,94,95,96]. This trend reflects a growing recognition that veterinary clinical decisions are influenced not only by diagnostic evidence and treatment efficacy but also by animal welfare, quality of life, owner preferences, available resources, and professional and ethical considerations. Future research should therefore explore how these diverse forms of information can be systematically integrated into clinical reasoning and decision-making. In this context, the integration of cognitive, behavioral, welfare-related, diagnostic, and evidence-based information may provide a more comprehensive framework for individualized veterinary care.

Generative AI is also beginning to be evaluated as a tool for directly supporting clinical reasoning in veterinary education [97]. This development parallels a broader shift in veterinary education, in which clinical reasoning is increasingly conceptualized not merely as an implicit clinical skill but as an explicit and teachable component of the curriculum [8,9,97]. Importantly, emerging evidence suggests that the educational value of generative AI depends on appropriate scaffolding and the ability of learners to critically evaluate AI-generated information rather than on AI assistance alone [97].

Ultimately, the future development of veterinary clinical reasoning will depend not only on advances in diagnostic science and cognitive theory but also on the ability to integrate these developments into a coherent, patient-centered decision-making process. The challenge for future veterinary medicine is therefore to determine how emerging technologies and expanding knowledge of animal cognition and welfare can augment, rather than replace, the clinician’s capacity for clinical reasoning and professional judgment.

Problem-Oriented Medical Records (POMR) and Evidence-Based Veterinary Medicine (EBVM) provide complementary frameworks for organizing clinical information and integrating scientific evidence into veterinary practice. Clinical reasoning extends beyond these functions by requiring clinicians to interpret patient-specific information, understand underlying disease processes, and determine appropriate actions within the clinical context. This review organized 15 theoretical frameworks into four categories according to their primary theoretical roles and demonstrated that these perspectives address complementary dimensions of clinical reasoning rather than mutually exclusive processes. Three representative ophthalmic scenarios further illustrated how different theoretical perspectives can contribute to pathophysiological interpretation and clinical decision-making. On this basis, PCCR is proposed as an integrative clinical framework in which pathophysiological interpretation links clinical information with assessment of severity and prognosis and subsequent clinical decision-making. PCCR is not proposed as a new cognitive theory or as a replacement for established approaches such as forward reasoning, illness scripts, POMR, EBVM, or the Clinical Reasoning Cycle. Its potential applicability beyond veterinary ophthalmology remains a hypothesis requiring empirical evaluation.

## 8. Conclusions

Problem-oriented medical records (POMRs) and evidence-based veterinary medicine (EBVM) provide complementary frameworks for systematically organizing clinical information and integrating scientifically grounded evidence into veterinary practice. However, clinical reasoning extends beyond the acquisition and organization of information, requiring clinicians to determine the most appropriate course of action for each individual case by integrating clinical findings with relevant knowledge, experience, and contextual factors. This review examined clinical reasoning in canine and feline ophthalmic practice, with particular emphasis on pathophysiological reasoning and the distinctive characteristics of the ophthalmic diagnostic process. Because the nature and availability of clinical information vary among individual cases, clinical reasoning must be adapted to the characteristics and diagnostic demands of each case. In this review, the theoretical perspectives underlying clinical reasoning were categorized into four groups encompassing 15 theoretical frameworks and these theoretical perspectives were applied to three representative clinical scenarios to illustrate how different cognitive processes may contribute to clinical reasoning. A clinical reasoning approach centered on pathophysiological inference is not limited to veterinary ophthalmology and may provide a broader conceptual framework for understanding, teaching, and evaluating clinical reasoning across other areas of veterinary medicine.

## Figures and Tables

**Table 1:** Theoretical frameworks relevant to clinical reasoning.

Theoretical Category	Theory	Overview	Ref.
Historical and Normative Theories of Reasoning	Logic and Normative Reasoning	A normative framework for evaluating the validity of inferences from premises to conclusions. Deduction, induction, and abduction provide complementary logical perspectives on how general knowledge and empirical observations may contribute to clinical judgments.	[25,26,27]
Foundational Theories of Modern Cognitive Science	Predictive Processing	A framework in which the brain generates predictions based on internal models and updates those models in response to discrepancies between predicted and incoming sensory information.	[28,29,30]
	Bayesian Brain Hypothesis	A framework in which perception and cognition are conceptualized as probabilistic inference that integrates prior knowledge with incoming evidence under uncertainty.	[31,32]
	Information Processing Approach	A cognitive framework describing how information is encoded, attended to, maintained in working memory, retrieved from long-term memory, integrated, and transformed into judgments or actions.	[33,34,35,36]
	Schema Theory	A framework proposing that knowledge is organized into structured representations that guide the interpretation and integration of new information.	[37]
	Mental Model Theory	A theory proposing that individuals construct internal representations of situations and use these representations to reason about possible states, causal relationships, and outcomes.	[38,39]
Theories of Clinical Reasoning and Decision-Making	Dual-Process Theory	A framework distinguishing relatively rapid and automatic Type 1 processing from slower and more deliberative Type 2 processing. Clinical reasoning may involve both intuitive pattern recognition and analytical reasoning.	[40,41,42]
	Illness Script Theory	A framework proposing that clinical knowledge is organized into illness scripts linking enabling conditions, pathophysiological mechanisms, and clinical consequences. The activation of relevant scripts can facilitate the interpretation of clinical findings and guide diagnostic reasoning.	[23,43,44]
	Hypothetico-Deductive Model	A model in which preliminary diagnostic hypotheses are formulated from initial clinical information and guide the selective collection of additional information used to test, refine, or reject those hypotheses.	[45,46]
	Forward Reasoning	A data-driven reasoning strategy that proceeds from observed findings through established causal or pathophysiological knowledge toward explanatory hypotheses and diagnostic conclusions. It has been associated with accurate expert performance in specific diagnostic tasks, although its relationship with expertise and task difficulty is complex.	[42,47,48]
	Naturalistic Decision-Making	A framework describing decision-making in complex, dynamic, and uncertain real-world environments, emphasizing experience, situation recognition, pattern recognition, and mental simulation.	[49,50]
	Situated Cognition	A perspective in which cognition and reasoning are shaped by interactions among individuals, activities, environments, social contexts, and material resources.	[51,52,53]
Theories of Clinical Education and Expertise	Metacognition	Awareness, monitoring, and regulation of one’s own cognitive processes. Metacognition can support the recognition of uncertainty, potential cognitive bias, and the need to reconsider diagnostic judgments.	[54,55,56]
	Reflective Practice	A deliberate process of examining experiences, actions, assumptions, and reasoning processes to derive insights that may improve future practice and professional development.	[56,57,58,59]
	Clinical Reasoning Cycle	An educational framework that conceptualizes clinical reasoning as an iterative process involving consideration of the patient situation, information collection, information processing, problem identification, goal setting, action, evaluation, and reflection.	[8,9,60]

**Table 2:** Examples of theoretical discussions in the clinical reasoning of a Shiba Inu with glaucoma.

Theoretical Category	Theory	Example of Theoretical Discussion
Historical and Normative Theories of Reasoning	Logic and Normative Reasoning	Elevated IOP alone does not establish primary glaucoma; findings such as breed, age, angle closure, pupillary changes, fundus abnormalities, lens status, and inflammatory signs are integrated to distinguish primary from secondary glaucoma and to assess the consistency of the diagnostic conclusion.
Foundational Theories of Modern Cognitive Science	Predictive Processing	Based on prior clinical knowledge of breed and age, primary glaucoma may be initially predicted. Concordant findings strengthen this working model, whereas unexpected findings such as uveitis or lens displacement would generate prediction errors and prompt model revision toward secondary glaucoma.
	Bayesian Brain Hypothesis	The Shiba Inu breed provides a relatively high prior probability of primary glaucoma. Subsequent findings, including ocular pain, elevated IOP, angle closure, pupillary abnormalities, and optic disc cupping, provide additional evidence that increases the posterior probability of primary angle-closure glaucoma.
	Information- Processing Approach/ Framework	Clinical information—including history, IOP, STT, pupillary responses, angle status, lens findings, and fundus changes—is sequentially collected, attended to, and integrated to determine the diagnosis, disease severity, prognosis, and treatment response.
	Schema Theory	The clinical findings can be organized within established diagnostic schemas, such as those associated with primary glaucoma and angle-closure glaucoma, allowing the case to be categorized according to the pattern of findings.
	Mental Model Theory	A causal model—angle closure → impaired aqueous outflow → elevated IOP → ocular and optic nerve damage—links the observed clinical findings to the underlying pathophysiology.
Theories of Clinical Reasoning and Decision-Making	Dual-Process Theory (DPT)	Type 1 processing may rapidly recognize glaucoma from a characteristic clinical presentation, whereas Type 2 processing can be used to evaluate primary versus secondary causes, visual function, disease severity, and treatment effectiveness.
	Illness Script Theory	The case can be compared with an illness script for canine primary angle-closure glaucoma, including predisposing characteristics, clinical presentation, elevated IOP, angle closure, pupillary abnormalities, and optic nerve damage.
	Hypothetico-Deductive Model	The preliminary hypothesis of primary angle-closure glaucoma is evaluated against the clinical examination findings, while alternative explanations are considered and additional findings are used to support, refine, or reject the hypothesis.
	Forward Reasoning	Observed findings are interpreted through established causal and pathophysiological knowledge to develop an explanatory model linking angle closure and impaired aqueous humor outflow to elevated IOP and subsequent ocular and optic nerve damage.
	Naturalistic Decision-Making	Because glaucomatous visual loss may be irreversible, management decisions must be made in a clinically consequential and uncertain context. Persistent pain and elevated IOP provide important cues prompting reassessment of the treatment strategy.
	Situated Cognition	Clinical decisions are shaped by the specific clinical context, including breed, age, disease duration, current treatment, clinical findings, and the circumstances of referral.
Theories of Clinical Education and Expertise	Metacognition	The clinician monitors potential cognitive biases, such as attributing glaucoma solely to breed or IOP, and reassesses treatment response, medication administration, adherence, disease stage, and angle closure.
	Reflective Practice	After the clinical encounter, the clinician can review whether visual function, differential diagnosis, angle assessment, and treatment response were adequately evaluated and use these insights to improve future practice.
	Clinical Reasoning Cycle	Consider the patient situation → collect and process clinical information → identify problems → establish goals → take action → evaluate outcomes → reflect and revise the reasoning process.

**Table 3:** Examples of theoretical discussions in the clinical reasoning of a Japanese domestic cat with descemetocele and aqueous humor leakage.

Theoretical Category	Theory	Example of Theoretical Discussion
Historical and Normative Theories of Reasoning	Logic and Normative Reasoning	A descemetocele indicates extreme stromal thinning but does not by itself prove perforation. A positive Seidel test and aqueous humor leakage provide direct evidence supporting corneal perforation.
Foundational Theories of Modern Cognitive Science	Predictive Processing	A severe corneal ulcer may initially generate a prediction that the lesion has progressed toward perforation. Confirmation of a positive Seidel test and aqueous humor leakage provides evidence that updates the clinical model toward established perforation.
	Bayesian Brain Hypothesis	The history and clinical findings generate competing probabilities for deep ulceration, infectious keratitis, stromal melting, and other severe corneal disease. Identification of a descemetocele and aqueous humor leakage provides additional evidence that increases the probability of corneal perforation.
	Information Processing Approach/ Framework	History, previous treatment, lesion size, ocular pain, ciliary hyperemia, visual responses, Seidel test, aqueous humor leakage, and STT are sequentially collected and integrated to assess lesion severity, visual function, and the need for urgent intervention.
	Schema Theory	The clinical findings can be organized within a progressive corneal disease schema, ranging from superficial ulceration through deep ulceration and stromal melting to descemetocele and perforation. Aqueous leakage indicates structural breakdown consistent with perforation.
	Mental Model Theory	A causal model—stromal melting → stromal thinning → exposure or bulging of Descemet’s membrane → full-thickness breakdown → aqueous leakage → loss of anterior chamber integrity—links the observed structural findings to their pathophysiological consequences.
Theories of Clinical Reasoning and Decision-Making	Dual-Process Theory (DPT)	Type 1 processing may rapidly recognize severe corneal disease from findings such as pain, blepharospasm, and a descemetocele, whereas Type 2 processing can analyze the cause and extent of corneal damage and evaluate the evidence for perforation.
	Illness Script Theory	Key findings—including ocular pain, blepharospasm, ciliary hyperemia, deep corneal damage, descemetocele, and aqueous leakage—can be compared with an illness script for severe corneal ulceration and perforation.
	Hypothetico-Deductive Model	The hypothesis of corneal perforation is evaluated against the descemetocele, positive Seidel test, aqueous humor leakage, and anterior chamber findings, while alternative explanations for the severe corneal lesion are considered.
	Forward Reasoning	Observed corneal findings are interpreted through established pathophysiological knowledge to anticipate structural failure and the consequences of aqueous humor leakage.
	Naturalistic Decision-Making	A positive Seidel test represents a critical clinical cue indicating corneal perforation and the need for urgent intervention, requiring prioritization of corneal and globe preservation even when the underlying cause of the corneal defect remains uncertain.
	Situated Cognition	Clinical decisions are shaped by the specific context, including the cat’s clinical status, previous treatment, duration of disease, and the circumstances of referral.
Theories of Clinical Education and Expertise	Metacognition	The clinician can question whether prior labeling as a “corneal ulcer” has biased the assessment and whether the presence of a descemetocele has led to under recognition of perforation; the lesion is therefore reassessed and the Seidel test is used to evaluate structural integrity.
	Reflective Practice	After the clinical encounter, the clinician can evaluate whether the deep lesion, risk of perforation, and need for referral were recognized appropriately and incorporate these observations into future assessment criteria.
	Clinical Reasoning Cycle	Consider the patient situation → collect and process clinical information → identify the deep corneal lesion → establish the clinical problem and goals → confirm structural compromise → initiate urgent treatment → evaluate the response → revise the treatment plan → reflect.

**Table 4:** Examples of theoretical discussions in the clinical reasoning of a Chihuahua with traumatic proptosis.

Theoretical Category	Theory	Example of Theoretical Discussion
Historical and Normative Theories of Reasoning	Logic and Normative Reasoning	The diagnosis of proptosis and the decision regarding globe preservation or enucleation represent separate clinical propositions. Globe preservation and visual preservation should therefore be assessed independently on the basis of injury severity and ocular findings.
Foundational Theories of Modern Cognitive Science	Predictive Processing	An initial prediction of traumatic proptosis is supported by the mechanism of injury and globe displacement. The discrepancy between preserved menace and dazzle responses and an absent direct pupillary light reflex provides additional information that should prompt reassessment of the clinical model and the interpretation of visual and pupillary pathway function.
	Bayesian Brain Hypothesis	The clinician can be understood as progressively updating the estimated probability of globe and visual preservation as evidence regarding injury duration, corneal damage, tissue injury, and visual responses accumulates.
	Information Processing Approach/ Framework	Clinical information is collected and integrated sequentially—from the injury mechanism and ocular findings to visual responses—to assess severity, prognosis, and treatment urgency.
	Schema Theory	The findings can be organized within a canine ocular trauma schema, with traumatic proptosis further characterized by globe displacement, corneal exposure, tissue damage, and the status of visual responses.
	Mental Model Theory	Trauma is interpreted through a causal model linking globe displacement and eyelid compression to impaired circulation, corneal exposure, and progressive tissue damage.
Theories of Clinical Reasoning and Decision-Making	Dual-Process Theory (DPT)	Type 1 processing may rapidly recognize proptosis, whereas Type 2 processing can evaluate injury duration, tissue damage, corneal status, and visual responses to determine the potential for globe and visual preservation.
	Illness Script Theory	The case can be compared with an illness script for traumatic proptosis in dogs, linking acute trauma, globe displacement, eyelid swelling, and corneal exposure to the diagnosis and its potential consequences.
	Hypothetico-Deductive Model	The hypothesis that the traumatic event has caused substantial ocular and periocular injury is evaluated against the examination findings, including globe displacement, corneal drying, conjunctival and scleral injury, and visual responses.
	Forward Reasoning	Observed traumatic findings are interpreted through established pathophysiological knowledge to develop hypotheses concerning vascular compromise, corneal exposure, tissue injury, and potential visual dysfunction, while anticipating possible progression of secondary damage.
	Naturalistic Decision-Making	Because traumatic proptosis requires prompt clinical management, critical cues such as globe displacement, corneal exposure, tissue injury, and visual responses must be rapidly integrated to prioritize corneal protection, stabilization, and assessment of the feasibility of globe preservation.
	Situated Cognition	Clinical decisions are shaped by the specific context, including the 48 h delay, patient size, prior treatment, current ocular status, and the circumstances of referral.
Theories of Clinical Education and Expertise	Metacognition	The clinician monitors potential cognitive biases, such as judging prognosis from the external appearance alone or interpreting an absent pupillary light reflex as definitive evidence of irreversible blindness and reassesses the findings in an integrated manner.
	Reflective Practice	After the clinical encounter, the clinician can review the timeliness of corneal protection, assessment of visual function, and the decision-making process concerning globe preservation and use these observations to improve future management.
	Clinical Reasoning Cycle	Consider the patient situation → collect and process clinical information → identify problems → establish goals → intervene → evaluate outcomes → revise the treatment plan → reflect.

## Data Availability

The data supporting the findings of this study are available from the corresponding author upon reasonable request.
